# Genetic transformation of einkorn (*Triticum monococcum L.* ssp. monococcum L.), a diploid cultivated wheat species

**DOI:** 10.1186/s12896-018-0477-3

**Published:** 2018-10-23

**Authors:** Dmitry Miroshnichenko, Danila Ashin, Alexander Pushin, Sergey Dolgov

**Affiliations:** 1 Institute of Basic Biological Problems RAS, Pushchino, Moscow Region Russian Federation; 2 Branch of Shemyakin and Ovchinnikov Institute of Bioorganic Chemistry RAS, Pushchino, Moscow Region Russian Federation; 3 All-Russia Research Institute of Agricultural Biotechnology, Moscow, Russian Federation

**Keywords:** Einkorn, Diploid wheat, Immature embryos, Particle bombardment, Stable transformation, Plant regeneration, Transgene inheritance, Herbicide resistance

## Abstract

**Background:**

Domesticated einkorn (*Triticum monococcum* L.) is one of the oldest cultivated cereal crops in the world. Its small genome size (~ 5.7 GB), low ploidy (2n = 2x = 14, A^m^A^m^) and high genetic polymorphism make this species very attractive for use as a diploid model for understanding the genomics and proteomics of *Triticeae.* Einkorn, however, is still a recalcitrant monocotyledonous species for the application of modern biotechnologies, including transgenesis. This paper reports the factors that may influence transgene delivery, integration, expression and inheritance in einkorn.

**Results:**

In this study, we report the successful genetic transformation of einkorn using biolistic-mediated DNA delivery. Immature embryo-derived tissues of spring einkorn were bombarded with a plasmid containing the reporter gene GFP (green fluorescent protein) driven by the rice actin promoter (*act1*) and the selectable *bar* gene (bialaphos resistance gene) driven by the maize ubiquitin promoter (*ubi1*). Adjustments to various parameters such as gas pressure, microcarrier size and developmental stage of target tissue were essential for successful transient and stable transformation. Bombarded einkorn tissues are recalcitrant to regenerating plants, but certain modifications of the culture medium have been shown to increase the production of transgenic events. In various experiments, independent transgenic plants were produced at frequencies ranging from 0.0 to 0.6%. Molecular analysis, marker gene expression and herbicide treatment demonstrated that *gfp*/*bar* genes were stably integrated into the einkorn genome and successfully inherited over several generations. The transgenes, as dominant loci, segregated in both Mendelian and non-Mendelian fashion due to multiple insertions. Fertile homozygous T_1_-T_2_ populations of transgenic einkorn that are resistant to herbicides were selected.

**Conclusion:**

To the best of our knowledge, this is the first report of the production of genetically modified einkorn plants. We believe that the results of our research could be a starting point for the application of the current biotechnological-based technologies, such as transgenesis and genome editing, to accelerate comparative functional genomics in einkorn and other cereals.

**Electronic supplementary material:**

The online version of this article (10.1186/s12896-018-0477-3) contains supplementary material, which is available to authorized users.

## Background

Domesticated einkorn, subspecies *Triticum monococcum L.* ssp. monococcum L. (2n = 14), is the only diploid cultivated species of wheat. It is considered one of the oldest crops, as it was domesticated around 9500 years ago, and it influenced the Neolithic Revolution [1]. For centuries, domesticated einkorn was largely used as both food and animal fodder in various areas, including the Middle East, the Balkans, the Caucasus, South Africa, and Central and Mediterranean Europe. Starting in the Bronze Age, it was gradually replaced by free-threshing cultivated polyploid durum and bread wheats. Due to its long cultivation history in various geographical and environmental areas *T. monococcum* is still considered an important genomic resource for the improvement of modern wheat, especially for resistance genes against various biotic and abiotic stresses [2]. Over the last decade, it has received interest as a health food because einkorn grains contain significantly more carotenoids, lutein and other nutritional substances than modern wheats [3].

The diploid nature of *T. monococcum* and its smaller genome size (~ 5.7 GB), together with its easy cultivation procedure, make this species an attractive and useful model for understanding the biology, genomics and proteomics of *Triticeae* [4]*.* The A^m^ genome of domesticated einkorn *T. monococcum* is closely related to the A^u^ genome of the wild einkorn *T. urartu*, the diploid donor of the A genome of the hexaploid (AABBDD) bread wheat (*T. aestivum*), which is currently the main cultivated wheat in the world. The large genome of bread wheat (approximately 17 GB), the presence of three closely related subgenomes with various inter-chromosome translocations and the high repeat contents have made modern wheat a more ‘difficult’ crop for breeding among cereals [5]. Comparative genomics and combinations of genomic resources from einkorn and other cultivated wheats can help discover new alleles and accelerate advancements in genome-based breeding of modern wheat.

Several modern biotechnological tools, including next-generation sequencing, quantitative trait loci, microarrays, transgenesis and genome editing, can be used to unlock the genomics of *Triticeae* [6, 7]. The function of genes in wheat has often been studied in model transgenic plants such as arabidopsis, tobacco and rice [6]. Similar studies on modern polyploid wheats faced many problems associated with variable transgene integration into the A, B, and D subgenomes, leading to complexities for transgene performance, frequent gene silencing and unstable expression in subsequent generations [8].

Considering the biologically similar properties of *T. monococcum* and other *Triticeae* species, domesticated einkorn provides an excellent opportunity to facilitate the functional characterization of genes in wheat using various transgenic approaches such as overexpression and down-regulation of endogenous genes using RNAi technology or gene editing. Genome editing technologies, in combination with transgenesis, could be used to mutate certain loci and generate several mutants. This can help facilitate the discovery of new genes and clarify the biological processes at the molecular and phenotypic levels [9].

Despite the substantial progress in biotechnological advancements for many cereals including hexaploid and tetraploid wheats, einkorn is still a recalcitrant monocotyledonous species for transgenesis. In the mid-1990s, several attempts were made to transfer foreign genes into the einkorn genome. Cultured cells of *T. monococcum* were the first of the *Triticeae* species that were used to demonstrate the successful direct gene transfer using a PEG-mediated approach [10]. Later, transient gene expression in einkorn cells was achieved using electroporation [11] and biolistic-mediated gene transfer [12, 13]. Although stably transformed einkorn cells were obtained using both electroporation [14] and PEG-mediated delivery [10], no transgenic plants were produced due to the lack of regeneration from protoplasts and cell suspensions. However, the routine application of genetic engineering methods for various scientific purposes is still hampered by the lack of available and highly efficient tissue culture systems in einkorn.

By using immature embryos as explants, we recently established an efficient plant regeneration protocol for domesticated einkorn [15]. The proper combination of plant growth regulators promoted the formation of embryogenic/morphogenic structures with a high frequency (90%) and more than 10 shoots per initial explant. Such efficacy is generally considered sufficient for research aiming to produce transgenic plants from cereal explants. The objective of the study reported here was to establish a protocol for incorporating transgenes into the einkorn genome using biolistic methods and to generate transgenic einkorn plants based on the previously developed regeneration protocol.

Particle bombardment is a simple, versatile and efficient way to achieve genetic transformation in *Triticeae*, especially in wheat [16]. It is also considered a more promising method for genome editing in wheat than are PEG- or agrobacterium-mediated approaches [17]. Since the bombardment parameters are species- and tissue-specific, the main objectives of this study were to adjust the physical and biological factors that influence the gene delivery into einkorn cells based on transient gene expression. Here, we report the optimization of biolistic delivery using a plasmid containing the green fluorescent protein gene. Since GFP is a non-destructive reporter system, it allows easy monitoring of both transient expression and the stable transformation events throughout all in vitro experimental stages [18]. In wheat, the GFP-based reporter system also significantly simplifies the analysis of the promoter specific transgene expression in tissues of mature plants [19, 20], efficiently documents the inheritance of introduced genes to progeny [21, 22] and provides the simple determination of transgene flow between cultivated GM and non-GM plants [23].

In this study, we describe the genetic transformation of einkorn based on the strategy of dual selection. In addition to the *gfp* gene, the introduced construct contains the selectable *bar* gene, which confers resistance to phosphinotricin (PPT), the active ingredient of glufosinate ammonium-based commercial herbicides. In wheat biotechnology, the *bar* gene is most commonly used for the production of transgenic plants compared to other selectable genes [16]. In this study and using this dual system (GFP + PPT), it was possible to obtain transgenic einkorn plants displaying the stable expression of foreign genes in various tissues and to confirm the transmission of the introduced genes into subsequent generations.

## Methods

### Plant material

A spring type of einkorn (*T. monococcum* L. ssp. monococcum L.), accession number PI 119435, kindly donated by Prof. Gennady I. Karlov and colleagues (All-Russia Research Institute of Agricultural Biotechnology), was used in this study. Seeds were planted in potted soil in a temperature-controlled glasshouse (18–28 °C) with additional lighting (up to 150 μmol m^− 2^ s^− 1^) to supplement the natural light and provide a 16 h photoperiod. Spikes were collected 12–14 days after anthesis, and immature seeds were isolated using forceps. Isolated seeds were surface-sterilized under continuous agitation for 2 min in 70% (*v*/v) ethanol and for 18 min in 16.5% (v/v) of commercial bleach (ACE laundry bleach) containing a few drops of Tween 20 before five-fold washing with sterile water. Slightly translucent, 0.75–1.5 mm embryos were dissected from caryopses under a stereomicroscope using sterile forceps and a scalpel and were placed scutellum-side up onto the callus induction media (Table 1).

**Table 1:** Composition of culture media for domesticated einkorn (*T.monococcum* L.) tissue culture^a^

Media (procedure)	Phytohormones, mg/l				Carbohydrates, %		PPT, mg/l	Duration period
	Dicamba	Daminozide	TDZ	GA_3_	Sucrose	Mannitol		
Callus induction medium basal	3	50	0.25		3			5–15 days
Callus induction medium modified	3	50	0.1		3			
Osmoticum medium	3	50			3	7.3		20 hours
Callus selection medium A	3	25			3		3	6 weeks
Callus selection medium B	3				3		3	
Callus proliferation medium	1.5				3		3	3 weeks
Differentiation medium	0.5				3		3	2–3 weeks
Regeneration medium					3		3	3–5 weeks
Rooting medium					2		2	2–4 weeks
Embryo germination medium				0.1	2			5–10 days

^a^MS medium consisted of MS basal salts and vitamins supplemented with 150 mg/l L-asparagine and 7 g/l agar was used as basic medium in all cases

### Plasmid

The plasmid psGFP-BAR [24], kindly provided by B.V. Conger, University of Tennessee, containing *sgfp* gene driven by the rice actin1 (*act1*) promoter and the *bar* gene expressed by the maize ubiquitin (*ubi*1) promoter (Fig. 3a) was used for transient and integrative transformation assays. The plasmid DNA was prepared using a commercially available GeneElute HP Plasmid Midiprep Kit (Sigma-Aldrich, USA). Prior to bombardment, the concentration of the plasmid DNA was adjusted to 1 μg/μl.

### Particle bombardment and transient assay

A particle inflow gun (PIG) [25] was used to deliver DNA-coated tungsten particles into the embryogenic tissues. Particles were prepared by vortexing in 100% ethanol, pelleting by centrifugation and washing three times with distilled water. The final microparticle concentration was adjusted to 100 μg/ml in sterile water. To adsorb DNA onto the tungsten particles, 5 μl of plasmid DNA was added to a 25 μl particle suspension and gently mixed for 1 min. Then, 25 μl of 2.5 M CaCl_2_ and 10 μl of 100 mM spermidine were promptly added to a particle-DNA mix. The final mixture was briefly vortexed and placed on ice for 3–5 min. Next, 50 μl of the supernatant was removed, and the remaining pellet was used for bombardment. For each shot, 2 μl of the dispersed pellet was loaded on the mesh of a 13 mm plastic swinney filter holder (PALL Gelman Laboratory, USA) that served as a carrier. Each plate was bombarded twice with 13 cm between the DNA expulsion point to the target tissue. Different helium pressures (65, 73, 80, 87 and 94 Psi) and tungsten particle sizes (0.4, 0.7 and 1.1 μm, Bio-Rad, USA) were studied in the transient assay. Two target tissues, i.e., embryos with pre-morphogenic structures (5 days of culturing on callus induction medium) and embryos with early morphogenic structures (10–15 days culturing on callus induction medium), were also investigated. The PIG operation was conducted as previously described [26]. Four hours before bombardment, explants were placed on an osmoticum medium (Table 1) in a 1.8-cm-diameter circle at the centre of 6-cm Petri dishes. The transient expression experiments were repeated three or four times, with two replicates of 12 explants in each replicate. For integrative transformation assays, one replicate contained 25–28 explants with early morphogenic structures.

### Selection and regeneration of putative transformants

After approximately 20 h of plasmolysis on an osmoticum medium, the bombarded explants were moved to callus selection media A or B (Table 1) and incubated with two subcultures for 6 weeks. The tissue with GFP fluorescence was separated from the non-embryogenic tissue and cultivated for 3 weeks on callus proliferation medium (Table 1). The surviving morphogenic tissues were placed for 2–3 weeks into the differentiation medium to induce the formation of shoot primordia and transferred to hormone-free regeneration medium. Explants regenerating putative transgenic plants were subcultured in regeneration medium (Table 1) for 10- to 15-day intervals until the plantlets reached approximately 3–4 cm in length. All indicated media were supplemented with 3 mg/l PPT and the einkorn cultures were kept in the dark at 25 ± 1 °C. Selected plantlets were transferred to rooting medium (Table 1) supplemented with 2 mg/l PPT and cultured under 16 h of light (100 μmol m^− 2^ s^− 1^) at 24 ± 2 °C. Rooted plants with a height of at least 10 cm were transplanted to soil as described [27].

### GFP monitoring

Visual screening was performed using a ZEISS SteREO Discovery.V12 microscope equipped with a PentaFluar S 120 vertical illuminator. Two commercially available filter sets 38 GFP BP (EX BP 470/40, BS FT 495, EM BP 525/50) and 57 GFP BP (EX BP 470/40, BS FT 495, EM LP 550) (Carl Zeiss MicroImaging GmbH, Germany) were used to examine transient and stable GFP expression. In the transient assay, the GFP-expressing cells were counted 24 h post-bombardment. Visual selection for GFP sectors began 3 weeks after bombardment and continued until plantlet regeneration.

### Molecular analysis

Total genomic DNA was extracted from leaf tissue at ear emergence in accordance with the method described [28]. Total RNA was isolated from the same material using the Aurum Total RNA Fatty and Fibrous Tissue kit (Bio-Rad, USA). RNase-free DNase I polymerase was used to degrade plant genomic DNA before the RT reaction. PCR analysis was carried out using specific primer pairs that were designed to amplify a 310-bp fragment of the *bar* gene (forward 5’-TGC ACC ATC GTC AAC CAC TA-3′; reverse 5’-ACA GCG ACC ACG CTC TTG AA-3′) and a 600-bp fragment of *gfp* gene (forward 5’-GCG ACG TAA ACG GCC ACA AG -3′; reverse 5’-CCA GCA GGA CCA TGT GTG ATC G -3′). To confirm the integration of the *bar* gene into the einkorn genome, genomic DNA (30 μg) was digested overnight at 37 °C with 60 U *Hind*III for Southern blot analysis*.* The fragments were separated on a 0.9% agarose gel and transferred to a positive-charged nylon membrane Hybond N+ (GE Healthcare, UK) using capillary blotting following the manufacturer’s instructions. The DNA probe was constructed using PCR with the plasmid psGFP-BAR as the template and the primers described above. The DNA probe was labelled with alkaline phosphatase using an Amersham Gene Image AlkPhos Direct Labelling and Detection System (GE Healthcare, UK). Prehybridization, hybridization (overnight at 60 °C) with the alkaline phosphatase-labeled probe, and subsequent washings of the membrane were carried out according to the AlkPhos Direct Labeling System protocol. Detection was achieved using CDP-Star detection reagent in accordance with the manufacturer’s directions (Amersham CDP-Star Detection reagent, GE Healthcare, UK).

### Segregation analysis

T_1_ and T_2_ progeny produced by self-pollination of primary T_0_ transgenic plants were assessed for transgene inheritance based on the GFP fluorescence of embryos/seedlings. To speed up the segregation analysis, T_1_-T_2_ embryos were isolated from kernels at soft dough with the same procedure as described for immature embryos, except the sterilization was done with 20% (*v*/v) commercial bleach for 22 min. Up to thirty embryos were placed scutellum-side down in petri dishes containing the germination medium (Table 1). Dishes were incubated for 3–7 days in the dark at 25 °C until the roots and coleoptiles reached a length of 0.5–1.5 cm to ensure the detection of GFP. The results of GFP fluorescence between observed and expected distributions against genetic ratios of 1:1, 3:1 and 15:1 were interpreted using the χ2 goodness of fit test (*P >* 0.05). To test herbicide resistance, seedlings were sprayed with 0.25% PPT solution. The results were observed after two weeks.

## Results

### Effect of bombardment parameters on transient expression

**Fig. 1:**
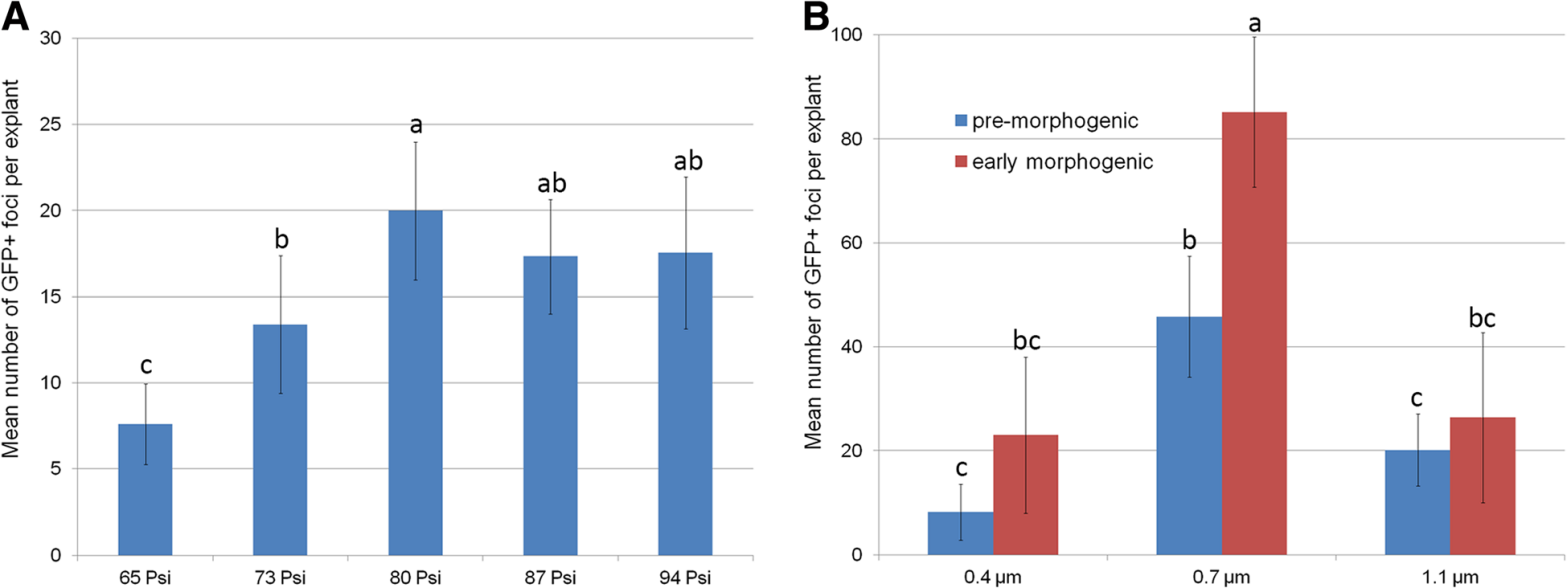
Effect of bombardment parameters and explant type on the level of transient GFP expression in einkorn cells. **A** Effect of helium pressure; embryos with pre-morphogenic structures were bombarded with 1.1 μm tungsten particles. **B** Relationship between tungsten particle size and explant type on levels of transient GFP expression; explants were bombarded using helium pressure of 80 Psi. Means with the same letter have no significant differences according to Duncan’s multiple range test (*P* < 0.05)

A transient GFP assay was used to optimize the efficiency of plasmid delivery into einkorn cells using PIG. Cultured embryos with pre-morphogenic structures (Additional file 1: Figure S1a) were bombarded using five helium pressures with 1.1 μm particles. An increase in helium pressure from 65 to 80 Psi resulted in a significant increase in transient GFP events (Fig. 1a). When the helium pressure was increased to 87 and 94 Psi, the number of observed GFP loci using these parameters did not differ significantly from the number observed at a pressure of 80 Psi. As a result, the helium pressure of 80 Psi was used in all transient and stable transformation experiments.

**Fig. 2:**
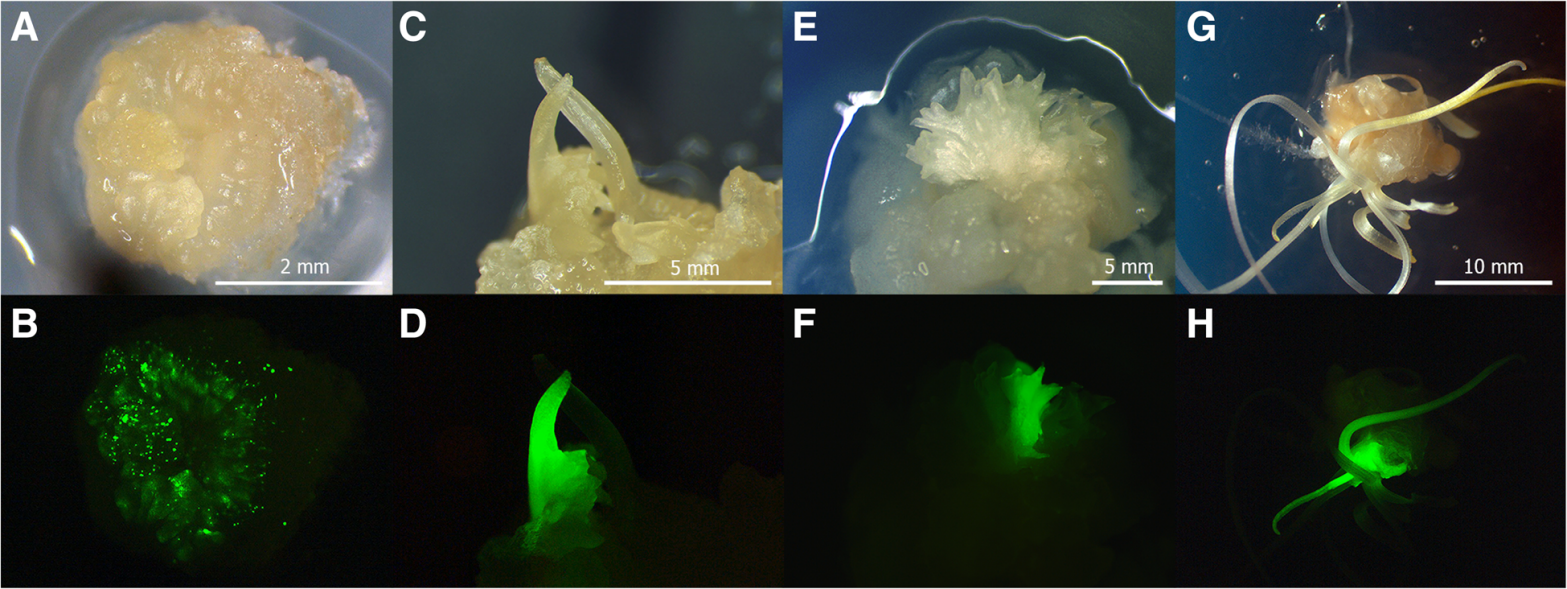
Selection of transgenic einkorn tissues after biolistic-mediated transformation with psGFP-BAR. **a**, **b** Transient GFP expression in morphogenic explant, 24 h after bombardment. **c**, **d** Formation of GFP-positive transgenic embryo, 55 days of culture. **e**, **f** Formation of chimerical (transgenic/untransgenic) cluster, 65 days of culture. **g**, **h** Development of transgenic regenerating plant, 75 days of culture. Tissues were photographed under white light (upper panel) or blue light (lower panel) using the GFP filter set (EX BP 470/40, BS FT 495, EM LP 550)

To determine the effect of particle size on the efficiency of transient expression, the einkorn explants were bombarded with equal weight of 0.4, 0.7 and 1.1 μm tungsten particles. Although transient *gfp* gene expression was observed in all bombarded explants, the number of visible loci was significantly affected by the particle size (Fig. 1b, Additional file 1: Figure S1). The smallest microprojectiles of 0.4 μm produced fewer GFP foci than did the 0.7 μm or 1.1 μm microprojectiles. Significantly higher numbers of GFP foci (on average 45 GFP spots) were observed per cultured embryo when the 0.7 μm tungsten particles were used as microprojectiles. Using the same experimental design, calli with early morphogenic structures (immature embryos cultivated 10–15 days on callus induction medium, Fig. 2a) were also bombarded. When the morphogenic callus was used as a target for bombardment, the number of green spots increased for all tested particle sizes (Fig. 1B). The greatest increase was observed when 0.7 μm particles were used. In this case, more than 80 green fluorescent spots (Fig. 2a) were found in approximately 80% of the explants bombarded. The bombardment of morphogenic callus with microprojectiles of 0.4 or 1.1 μm produced approximately the same number of GFP foci (20–25 green spots). The ANOVA analysis, however, indicated that the number of observed foci using the callus did not differ significantly from that observed when using cultured embryos for both 0.4 and 1.1 μm particles (*P* < 0.05).

Therefore, we decided to use a helium pressure of 80 Psi, 0.7 μm tungsten particles and explants with early morphogenic structures as bombardment targets in the subsequent experiments.

### Selection of transgenic tissues and regeneration of einkorn transgenic T_0_ plants

A combination GFP and herbicide selection system was used in our study to select transgenic plants. Transient expression of the *gfp* gene was easily detected in einkorn cells during the first ten days after bombardment. Then GFP fluorescence intensity started to decline, indicating that the observed expression was a result of non-integrated DNA. Some of the callus pieces, however, still demonstrated detectable fluorescence throughout the subsequent subcultures. Every three weeks, fluorescent tissues surviving on the PPT selection media were separated from non-fluorescent callus and transferred to the fresh selection media to induce the formation of putative transformants (Fig. 2b).

The production of stable transformants was first attempted using morphogenic explants produced on callus induction medium containing Dicamba, Daminozide and TDZ. In preliminary experiments, attempts to generate transgenic plants failed because of significant browning and the death of both transgenic and non-transgenic structures in the first subcultures on the selection medium supplemented with PPT. To overcome the excessive browning of einkorn explants, various combinations of induction and selection media were further used in the stable transformation experiments (Table 1).

In five independent experimental sets, a total of 1076 explants were bombarded with the psGFP-BAR plasmid. Half of bombarded morphogenic calli were induced using a lower level of TDZ. This approach reduced the extent of tissue browning and allowed the selection of an increased number of morphogenic structures with stable GFP expression (15 vs. 5) (Table 2). While the callus formation and morphogenic capacity of bombarded tissues was generally comparable with non-bombarded einkorn tissues, GFP positive transgenic clusters exhibited slower growth and a low frequency of morphogenesis. Regardless of which hormonal composition was used to induce morphogenic explants, the regeneration of whole plants from transgenic morphogenic clusters remained problematic.

**Table 2:** Impact of medium composition on genetic transformation frequency of domesticated einkorn *T.monococcum* L.

Induction medium	Selection medium	No. of bombarded explants	No. of GFP positive morphogenic structures survived the selection	No. of regenerated plantlets that survived the selection	Number of independent transgenic plants (PCR+)	Transformation efficiency (%)
Basal^a^	A^c^	265	3	2	1	0.38
	B^d^	271	2	1	0	0.00
	Total/Average	**534**	**5**	**3**	**1**	**0.19**
Modified^b^	A^c^	245	8	2	1	0.41
	B^d^	295	7	2	2	0.68
	Total/Average	**540**	**15**	**4**	**3**	**0.56**

^a^MS basal salts and vitamins, 150 mg/l L-asparagine, 3 mg/l Dicamba, 50 mg/l Daminozide, 0.25 mg/l TDZ

^b^MS basal salts and vitamins, 150 mg/l L-asparagine, 3 mg/l Dicamba, 50 mg/l Daminozide, 0.1 mg/l TDZ

^c^MS basal salts and vitamins, 150 mg/l L-asparagine, 3 mg/l Dicamba, 25 mg/l Daminozide, 3 mg/l PPT

^d^MS basal salts and vitamins, 150 mg/l L-asparagine, 3 mg/l Dicamba, 3 mg/l PPT

Since one of the hormonal components used for einkorn morphogenic callus induction is Daminozide, a known plant growth retardant [29], some of the bombarded explants were subcultured on the selection medium omitting this plant growth regulator. The removal of Daminozide from the callus selection medium did not significantly affect the plant formation, as approximately the same number of plantlets (4 vs. 3) were produced (Table 2).

The duration between the initial bombardment and the formation of GFP-positive plantlets ranged from 12 to 17 weeks. In some cases, transgenic clusters providing protection from PPT were developed along with non-transgenic morphogenic clusters (Fig. 2c). These clusters were able to produce non-transgenic plantlets lacking GFP fluorescence, mainly at the regeneration stage (Fig. 2d). Non-transgenic ‘escapes’ could be easily removed during the ‘dark’ cultivation steps using a stereomicroscope with continual visual GFP observation. When the plates with the clusters of regenerants were transferred to light, screening by GFP fluorescence became difficult due to the increase in chlorophyll content.

At the end of experiments, seven putative transgenic plantlets derived from independent explants were selected. Plantlets produced roots in the selection rooting medium under the light in magenta boxes and were transferred to the greenhouse. Six independent plants easily passed the adaptation to the ex vitro condition, but one independent plant did not recover after the transfer and died.

### Characterization of primary (T_0_) transgenic plants

**Fig. 3:**
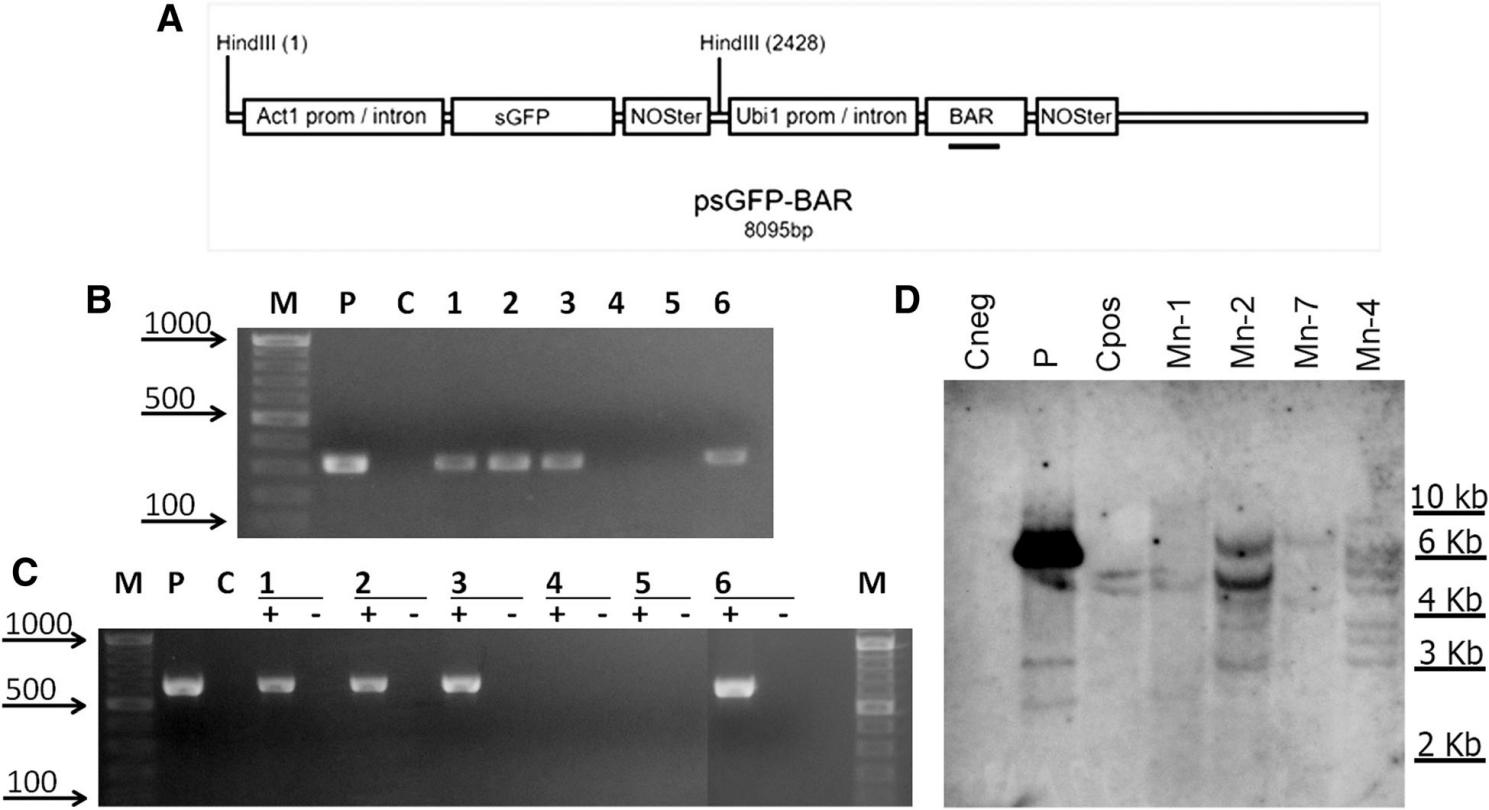
Molecular analyses of putative transgenic einkorn plants. **a** Schematic diagrams of psGFP-BAR vector used for einkorn transformation. Thick bar represents the positions of *bar* probe. **b**, **c** Primary plants produced within experiment were analyzed for the presence of *bar* gene by PCR amplification (b) and GFP expression by RT-PCR(c); Lane M, DNA ladder as molecular weight marker; Lane P, psGFP-BAR; Lane C, untransformed einkorn plant; Lane 1–6 represents putative transgenic plants Mn-1, Mn-2, Mn-4, Mn-5, Mn-6, Mn-7, respectively; +, a sample with addition of reverse transcriptase; −, a same sample without addition of reverse transcriptase. **d** Southern blot analyses of genomic DNAs from PCR positive primary transgenic plants; genomic as well as plasmid control DNA was digested with *Hind*III and the membrane was probed using the 310-bp *bar* probe; Lane P, psGFP-BAR (1.0 ng); Lane Cneg, negative control representing DNA from untransformed einkorn plant; Cpos, positive control, consisting of DNA from transgenic bread wheat plant with two transgene insertions; Lines Mn-1, Mn-2, Mn-4, Mn-7, primary transgenic einkorn plants

When the putative transgenic plantlets grew sufficiently to collect leaf samples, total DNA and RNA were isolated and subjected to PCR analysis. The PCR results showed the presence of the *bar* gene in the genome of four putative transgenic plants (Fig. 3b). The gene-specific primer combinations used for RT-PCR analysis showed that *gfp* gene was integrated and successfully transcribed in all *bar* positive T_0_ plants (Fig. 3c). Plants that were proven to be PCR-positive were further analysed using Southern blot for transgene integration. Total DNA was digested with *Hind*III to cleave the psBAR-GFP vector from the outside of the *bar* gene and hybridized with a *bar* probe (Fig. 3a). Each hybridizing band can be interpreted as a separate integration event. As expected, no detectable hybridization was observed in the untransformed einkorn plants, whereas primary transgenic plants exhibited different integration patterns (Fig. 3d) with various band sizes. Only multiple insertions of the *bar* gene (from two to seven) were observed in the resulting transgenic einkorn plants.

According to molecular analysis, the overall transformation efficiency of einkorn (independent transgenic plants per bombarded explants) was 0.37%. When the transformation frequencies over all sets of experiments were calculated using callus selection media without TDZ, transgenic plants were generated at a frequency of 0.56%. When TDZ was present in callus selection media, a lower transformation frequency of 0.19% was achieved.

**Fig. 4:**
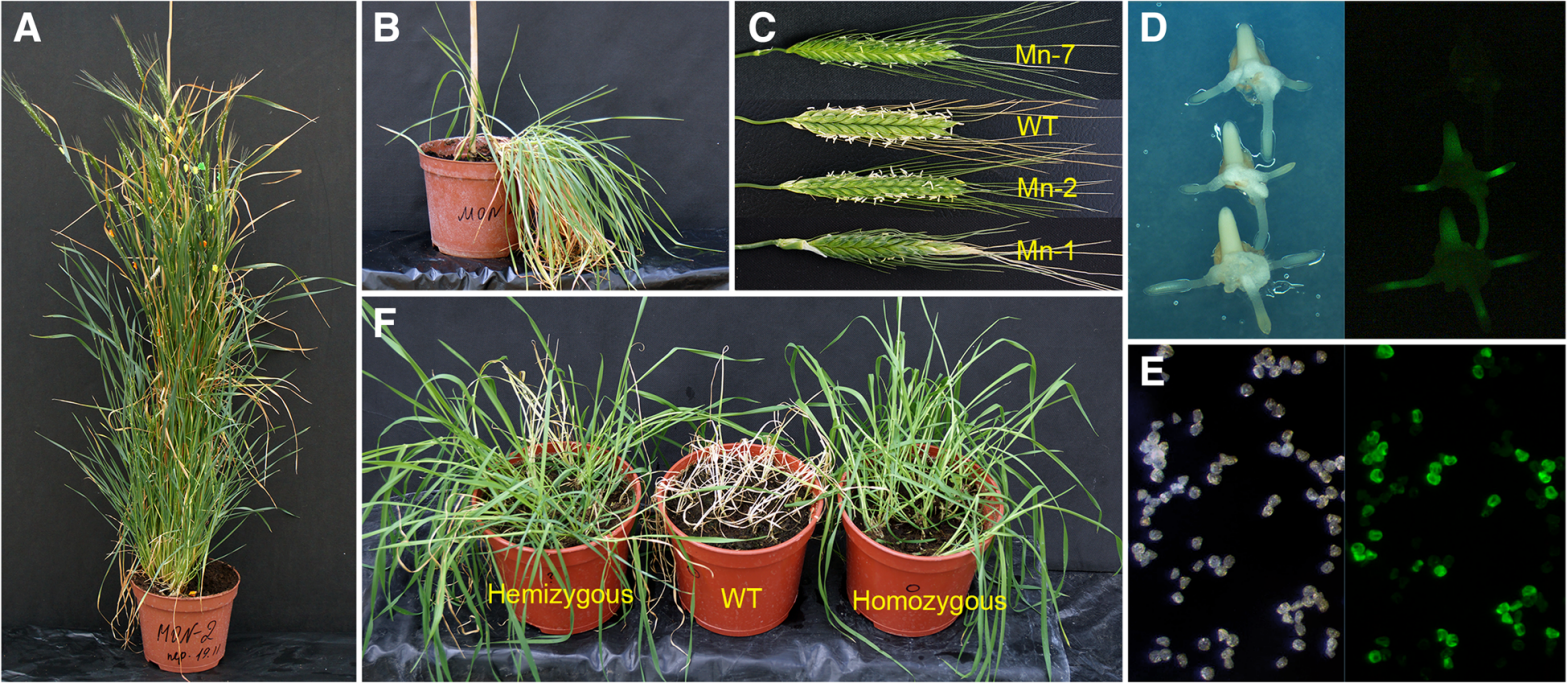
Genetic transformation of einkorn. **a** Tiller set of primary transgenic plant Mn-2. **b** Morphology of primary transgenic plant Mn-4. **c** Morphological comparison of tillers produced by primary transgenic einkorn plants and untransgenic tiller (WT). **d** Inheritance of GFP expression in excised T_1_ embryos of primary transgenic plant Mn-2 cultured in vitro, 5 days of culture; left panel: white light, right panel: the GFP filter set (EX BP 470/40, BS FT 495, EM LP 550). **e** GFP expression in pollen of primary transgenic plant Mn-7, left panel: white light, right panel: the GFP filter set (EX BP 470/40, BS FT 495, EM LP 550). **f** Herbicide resistance in non-transgenic einkorn (middle) and hemizygous (left) and homozygous (right) T_2_ populations of transgenic T_1_ plants resulted from the self pollination of primary transgenic einkorn plant Mn-2, photographed 2 weeks after the spray with 0.25% PPT

Among the primary transformants, the growth of the primary transgenic plant Mn-4 was generally reduced compared to those of other transgenic plants (Fig. 4a and b). This transgenic event produced a limited number of tillers, which were stunted and sterile. Other plants displayed normal tiller setting (Fig. 4c). Pollen of the all primary transgenic plants demonstrated clear GFP expression (Fig. 4e). Transgenic plant Mn-1 demonstrated partial fertility, as only 13 seeds were formed. Mn-2 and Mn-7 were similar to those of seed-derived control plants; both primary transgenic plants displayed a normal seed set ranging from 54 (Mn-7) to 338 seeds/plant (Mn-2).

### Transgene inheritance in T_1_-T_2_ progenies

To confirm that the transgene was transmitted to the next generation, seedlings of self-pollinated transgenic plants were examined for *gfp* expression. Screening the T_1_-T_2_ populations of Mn-1, Mn-2 and Mn-7 showed the presence of the GFP in all tested populations. The expression of *gfp* was visually detected in immature embryos during seedling germination, coleoptile extension and root development (Fig. 4d). The level of *gfp* expression varied between individual embryos and cell types. Generally, young, actively dividing and growing tissues, such as the meristems of the root tips, demonstrated the most intense signal. This allowed the easy distribution of the T_1_-T_2_ embryos into segregating and non-segregating offspring (Fig. 4d). Segregation studies based on the GFP fluorescence showed both Mendelian and non-Mendelian inheritance of transgenes in the progenies (Table 3).

**Table 3:** Segregation analysis of GFP expression in T_1_ and T_2_ progeny of transgenic plants of domesticated einkorn (*T. monococcum* L.)

T_0_ parent plant (T_1_ progeny plant)	Number of plants tested	Observed GFP segregation ratio (positive:negative)	χ^2^ value for the expected segregation ratio (positive:negative)		
			1:1	3:1	15:1
T_1_ plants					
Mn-1	13	8:5	0.69^a^	1.26^a^	23.02
Mn-2	179	125:47	35.37	0.50^a^	130.39
Mn-4	Sterile plant				
Mn-7	30	19:11	2.13^a^	2.18^a^	47.37
T_2_ plants					
Mn-1(1)	5	3:2	0.20^a^	0.6^a^	9.72
Mn-1(2)	6	6:0	Homozygous plants		
Mn-2(3)	15	15:0	Homozygous plants		
Mn-2(4)	47	36:11	13.30	0.06^a^	23.60
Mn-2(7)	34	24:10	5.77	0.35^a^	31.13
Mn-2(10)	18	18:0	Homozygous plants		
Mn-7(2)	12	6:6	0.00^a^	4.00	39.2
Mn-7(3)	33	16:17	0.03^a^	12.38	115.40
Mn-7(4)	18	10:8	0.22^a^	3.63^a^	44.82

^a^ if χ^2^ value is above 3.84 (*P* > 0.05) the observed segregation ratio is not significantly different from the expected ratio

Despite the multiple transgenic inserts, seedlings of the primary plant Mn-2 showed a segregation ratio of 125:47, which generally fits a 3:1 Mendelian segregation pattern (χ2 = 0.50; *P* > 0.05), indicating that the *gfp* gene behaved like a single dominant allele. The inheritance of GFP expression in the progenies of transgenic plant Mn-7, which had two transgene insertions, did not fit the expected ratio 15:1 that was generally observed for the two single dominant allele segregations. Seedlings of Mn-7 showed an aberrant segregation ratio close to 1:1 (Table 3). The same non-Mendelian inheritance of GFP expression lower than 3:1 was also found in the progeny of the primary transgenic plant Mn-1.

To further analyse the transmission of the transgenes to the T_2_ offspring, several T_1_ plants resulting from the primary T_0_ parents Mn-1, Mn-2 and Mn-7 were selected and self-fertilized to give a segregating population of T_2_ plants. Studies at the expression level (GFP fluorescence, herbicide spraying) followed by PCR analysis for the presence of the *gfp* gene found no differences in segregation patterns for different populations derived from the same T_0_ transgenic parent (Table 3). As expected, both hemizygous and homozygous plants were found. In hemizygous T_1_ plants such as Mn-2(4) and Mn-2(7), the GFP expression was present in 60/81 (74%) of the progeny and absent in 21/81 (26%) of the progeny (Table 3). This was not significantly different from the 3:1 ratio expected for a single dominant allele that was previously observed in T_1_ population of the selfed parent Mn-2. In T_1_ plants that were homozygous for the transgene, such as Mn-2(3) and Mn-2(10), both the GFP expression and *bar* gene inheritance (Table 3, Additional file 2: Figure S2) were present in all tested progeny seedlings. The results of herbicide resistance assays (Fig. 4f) were observed to be consistent with those of molecular analysis and GFP expression. Putative homozygous T_2_ plants were also found in the Mn-1(2) plant (Table 3). The limited number of seeds produced by the progenies of this parent does not allow us to insist that Mn-1(2) progenies are, in fact, homozygous. The primary transgenic plant Mn-7 failed to produce GFP expressing homozygous T_1_ progeny. The offspring of self-pollinated T_1_ plants exhibited the same 1:1 segregation pattern as the self-pollinated parent plant Mn-7 did (Table 3, Additional file 2: Figure S2), indicating the genetic stability of the inheritance pattern over generations.

## Discussion

The limited progress in einkorn genetic modification using various biotechnology approaches is mainly due to difficulties associated with low tissue culture efficiency. In this study based on the protocol that we previously developed, the genetic transformation of domesticated einkorn was achieved using particle bombardment.

There is general agreement that the efficiency of biolistic-mediated genetic transformation of cereals is influenced by several physical and biological factors [30, 31]. In *Triticeae*, numerous systematic studies of the parameters affecting the efficiencies of gene transfer were undertaken in polyploid wheat [21, 22, 32–35], barley [36, 37] and rye [38], although no studies have been published for diploid einkorn. Since the differences in cell size, cell division and morphogenesis patterns between einkorn and other cereals may affect the efficiency of particle penetration, three keys factors, i.e., helium pressure, particle size and explant type, were explored in this study. Their impact on the transient einkorn transformation was investigated using a ‘Low helium pressure’ device such as a Particle Inflow Gun (PIG). This microprojectile accelerator was designed by Finer et al. [25] and is well known as a low-cost alternative to a commercial ‘high helium pressure’ PDS 1000/He device (BioRad, Hercules, CA, USA). Generally, there is no ‘recommended standard’ for PIG acceleration pressures, and the optimal values vary greatly from 40 Psi (cell suspension) [39] to 320 Psi (mature embryos) [40] in the published literature for various species. In the case of einkorn, the best plasmid delivery into morphogenic tissues was found when the gas acceleration pressure was adjusted to 80–94 Psi. This result is consistent with studies performed in embryogenic callus of hexaploid wheat [41, 42] and some grasses [43], where similar biological and physical parameters were used. In other reports on cereals such as sorghum [40], pearl millet [44] and maize [45], the substantial increase in the efficiency of gene transfer occurred when the higher values (150–319 Psi) were used. In einkorn, these high acceleration pressures damaged explants, and they were not able to produce plants. This indicated a clear species-dependent requirement for this parameter.

Physical biolistic parameters such as particle size also greatly influenced the efficiency of transient transformation of einkorn tissues. It was evident that ‘medium sized’ particles of 0.7 μm were more effective in the transient delivery of the plasmid into einkorn cells than were bigger or smaller ones. It is noteworthy that this trend was observed for morphogenic einkorn tissues of different developmental stages. Generally, the beneficial effect of smaller particle size on transformation efficiency is attributed to less sustained damage being inflicted on bombarded cells than by the larger ones [34]. The observation that larger microprojecticles are more effective for plasmid delivery into cells compared to smaller and ‘lighter’ projectiles is attributed to the achievement of the appropriate kinetic energy [46]. Considering the large number of published reports on biolistic transformation, it is not surprising that somewhat controversial data could be found, even for the same species. In polyploid wheats, a species closely related to einkorn, the most effective particle size for plasmid delivery was in the range of 0.6–1.1 μm. A comparative study of [22] higher transient expression observed polyploid wheat explants being bombarded with 1.0 mm particles. Micro-carriers of this size, both gold and tungsten, were used repeatedly for successful generation of transgenic wheat plants [47–49]. In reports on plants similar to einkorn, the use of a ‘lighter’ particle size (0.6–0.7 μm) resulted in a significant increase in transient/stable wheat transformation [21, 34, 50] and has been used in various studies to introduce various foreign genes into the bread wheat genome. In some cases, the size of the microcarriers did not have a significant effect on the biolistic delivery of DNA into wheat explants, so a mixture of particles of different sizes was recommended as the best approach [33].

The successful survival of the bombarded explants is a serious problem in obtaining transgenic plants since the apparent repression of regeneration processes occurs after penetration of microcarriers into cells, especially when the tungsten particles were used [50]. For this reason, the optimum stage of explant development is another important factor that, similar to helium pressure and particle size, influences ability of microcarriers to penetrate the cell wall without damaging target tissues and can determine the possibility to retain regeneration potential.

In einkorn, scutellar tissues that were cultured for short times on induction medium were not optimal targets because of the lower number of cells were susceptible to biolistic DNA delivery. These explants seemed to be more damaged after bombardment and displayed clear reduction in morphogenic ability. In contrast, einkorn explants cultivated for longer periods of time on the callus induction medium were more susceptible to plasmid delivery and more resistant to remote effects of particle penetration. Due to the formation of early morphogenic structures before bombardment, these explants were still able to produce many regenerable clusters after particle delivery. Nonetheless, the realization of the morphogenic potential of einkorn explants was still problematic in subsequent subcultures and further optimisation work is required.

In the preliminary experiments, when transformed/untransformed cells were subjected to selection stress by a range of PPT concentrations (1–5 mg/l), significant browning and death of both transgenic and non-transgenic structures were observed. This blocked the process of morphogenesis and plant regeneration, including tissues that demonstrated stable GFP expression. Because the high morphogenic competence in cultivated einkorn tissues highly correlated with a suitable content of three phytohormones of various biological activity (Dicamba+TDZ + Daminozide), we supposed that the addition of PPT had a supplemental toxic effect due to the interaction with one of the substances. Two strategies are studied here to overcome this problem: the modification of the induction medium by reducing the TDZ level and the removal of Daminozide from the selection medium. Additionally, a relatively low PPT concentration (3 mg/l) was used for tissue selection.

Previously, we found that an excessive concentration of Daminozide, which is widely used in agriculture as a plant growth retardant [29], negatively influenced the plant regeneration in the tissue culture of bread wheat and einkorn [15, 51]. Given this experience, we assumed that its removal from the selection medium could benefit plantlet development. However, this approach did not improve the formation of the plants from transgenic morphogenic clusters of einkorn. A positive effect on the survival of transgenic morphogenic clusters was found after a significant reduction of the TDZ level in the induction medium followed by its complete removal from the selection medium. With this approach, most of the transgenic einkorn plants were produced. It should be noted that some browning of einkorn explants was earlier observed under nonselective conditions, especially when higher concentrations of TDZ were used to stimulate a morphogenic response [15]. Therefore, it is concluded that the difficulty in recovering transformed plants in einkorn was related to the remote toxic effect of TDZ during PPT selection. This observation can be attributed to several aspects of the complex nature of TDZ activity, which was originally developed as a cotton defoliant [52]. It is well documented that TDZ activity is very persistent in cultured tissues, even after the removal of this cytokinin-like substance from the medium [53]. Since the system for plant regeneration in einkorn is significantly different from other known methods described in the literature for cereals, further media manipulation with various cytokinin-like substances can be considered an important variable for the successful control of transgenic plant regeneration.

In different cereal and grass species, the PPT-based resistance conferred by the *bar* gene was shown to be more effective for the recovery of stable transgenic plants than were other selectable marker genes [54, 55]. In Triticeae species, the inefficient elimination of untransformed events was a common problem for PPT selection. In rye, when PPT was applied during the callus selection stage, the suppression of shoot and root regeneration was not observed [38]. Transformation of bread and durum wheats also largely suffered from a high frequency of PPT-resistant escapes (30–90%) in those studies, where high concentrations (5–15 mg/l) were used for selection [33, 47, 48, 56]. In barley, approximately 4–50% of the surviving plants were identified as escapes using moderate concentrations of PPT-based selective agent [57, 58]. Unlike the abovementioned species, the attempts to obtain PPT-resistant transgenic plants of sorghum failed because of high phenolic release by sorghum tissues in early stage of regeneration [59]. In einkorn, the relatively low selective pressure of PPT allowed the generation of transgenic plants with a frequency of 0.38–0.68% but did not completely prevent the formation of untransformed escapes. Nevertheless, the observed escape rate (~ 33%) is comparable to those of previous investigations involving various elite varieties of bread and durum wheat, which are closely related species. Considering the drawbacks of using PPT in this study, an alternative selection scheme that includes antibiotic resistance or substrate analogues as selective agents must be further analysed to enhance the success of genetic transformation in einkorn.

The transgenic events of einkorn produced fertile plants that expressed both the *bar* and *gfp* genes and successfully transmitted the introduced sequences to T_1_-T_2_ progenies. Our results indicate that *gfp* gene was a reliable indicator for controlling genetic transformation of einkorn. At various stages, GFP expression was easily detected in einkorn tissues and organs with low amounts of chlorophyll such as cells, calli, plantlets, roots, pollen grains, embryos and germinating seeds (Fig. 2 and Fig. 4d and e). Additionally, it was very helpful in identifying transgenic progeny after the self-pollination of transgenic plants.

Both Mendelian and non-Mendelian segregation patterns of the transgenes in the einkorn transgenic lines were observed. One event showed no significant deviation from Mendelian 3:1 ratio (Table 3). It is noteworthy that the number of transgenic inserts in this event (as estimated from Southern blot) was greater than one (Fig. 4), indicating that these copies were integrated into one locus. Other transgenic events displayed distorted ratios of transgene expression close to 1:1 (transgenic/non-transgenic). Similar non-Mendelian fashion of transgene segregation has been frequently observed in the progeny of transgenic cereals produced either by particle bombardment or *Agrobacterium*-mediated transformation. Several hypotheses were proposed to explain this phenomenon. There was a suggestion that the 1:1 segregation ratio could be due to aberrant gamete development [60]. The lack of fit to expected segregation ratios may be due to factors such as transgene silencing, position effects, multiple transgene copies, DNA rearrangements, promoter methylation and genetic chimerism of the tissues produced in the primary transgenic plant [61]. Since both T_0_ → T_1_ and T_1_ → T_2_ progenies displayed the same 1:1 ratio of transgene inheritance, chimerism can hardly be considered a possible reason for non-Mendelian segregation. Because all of the independent transgenic events of einkorn yielded more than one insertion of transgene, further analysis is required to determine the mechanism responsible for segregation distortion. Despite the unpredictable correlation between the numbers of inserts and patterns of transgene inheritance, the biolistic-mediated approach described here allowed us to obtain fertile T_2_ homozygous populations of einkorn. The developed plants displayed both good transmission of *bar/gfp* transgenes and reliable resistance to herbicide treatment.

## Conclusion

This is the first study to describe the successful production of transgenic plants in einkorn (*Triticum monococcum* L.), a recalcitrant diploid wheat species. Although the transformation efficiency calculated from transgenic event-producing experiments were approximately 0.5%, it is applicable for introducing transgenes into the einkorn genome for practical interests. This efficiency is lower than those reported in the most successful biolistic transformation studies for certain elite varieties of other *Triticeae* species such as barley [36], bread wheat [22, 33, 62], but it is comparable with the transformation efficiency of rye and durum wheat [38, 49]. The results presented here indicate that the low plant transformation frequency in einkorn is not due to inability to stably transform the cultured morphogenic tissues but the difficulty in regenerating transformed plants during selection. Considering that the initial frequency of transformation in many pilot biolistic transformation studies of polyploid wheat also fluctuated by approximately 0.01–1% [32, 41, 47, 48, 56], further examination of additional factors is expected to result in the more efficient production of transgenic einkorn events.

## Additional files

Additional file 1: **Figure S1.** Effect of tungsten particle size on the efficiency of plasmid delivery into cells of einkorn explants. Explants were bombarded with equal weight of 0.4 μm (**c**, **d**), 0.7 μm (**e**, **f**) and 1.1 μm (**g**, **h**) tungsten particle using helium pressure of 80 Psi. Untreated (**a**, **b**) and bombarded explants were analysed for the transient GFP expression 24 h after bombardment. Tissues were photographed under white light (**a**, **c**, **e**, **g**) or blue light (**b**, **d**, **f**, **h**) using the GFP filter set (EX BP 470/40, BS FT 495, EM LP 550). (PNG 3830 kb)

Additional file 2: **Figure S2.** Inheritance of the *gfp* gene in T_2_ progeny of transgenic einkorn, assessed by PCR. Examples of a 1:1 ratio segregation (T_2_ progeny of T_1_ plants Mn-7(3) and Mn-7(4)) and homozygous transgene inheritance (T_2_ progeny of T_1_ plant Mn-2(10)); Lane P, plasmid control, Lane C, untransformed einkorn plant; Lane numbers 1–23, T_2_ progeny plants. (PNG 421 kb)

## Abbreviations

*act1*: Rice actin promoter
ANOVA: Analysis of variance
*bar*: Bialaphos resistance gene
GFP: Green fluorescent protein
PIG: Particle inflow gun
PPT: Phosphinothricin
Psi: Pound per square inch
TDZ: *N*-phenyl-*N′*-(1,2,3-thiadiazol-5-yl)urea (thidiazuron)
*ubi1*: Maize ubiquitin promoter
