# Supplementary figures and images for: Genetic transformation of einkorn (Triticum monococcum L. ssp. monococcum L.), a diploid cultivated wheat species
Source: BMC Biotechnol. 2018 Oct 23;18:68. doi: 10.1186/s12896-018-0477-3 (PMC6199808; doi:10.1186/s12896-018-0477-3)

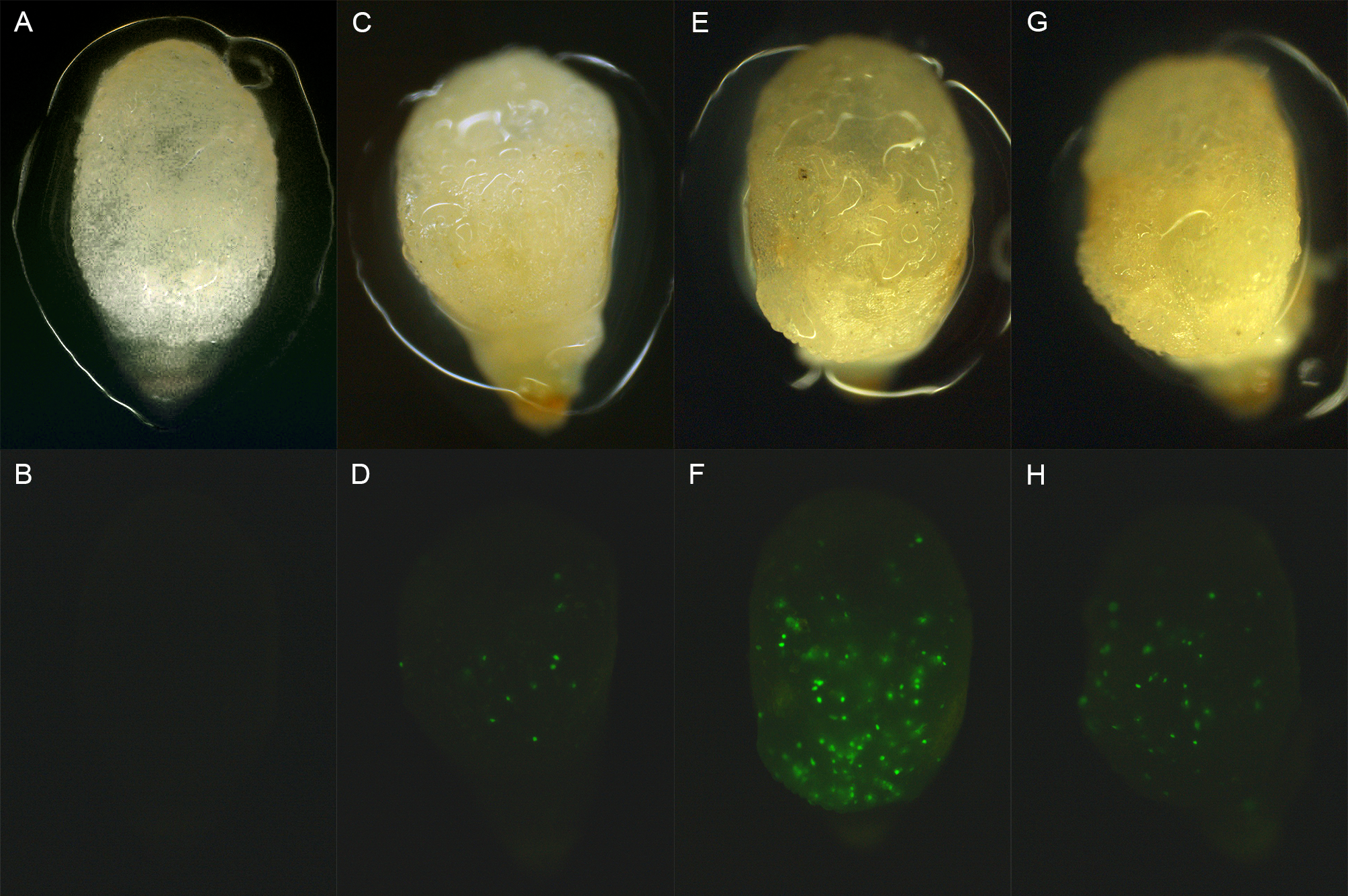

Supplement: Supplementary file 1 — Figure S1. Effect of tungsten particle size on the efficiency of plasmid delivery into cells of einkorn explants. Explants were bombarded with equal weight of 0.4 μm (c, d), 0.7 μm (e, f) and 1.1 μm (g, h) tungsten particle using helium pressure of 80 Psi. Untreated (a, b) and bombarded explants were analysed for the transient GFP expression 24 h after bombardment. Tissues were photographed under white light (a, c, e, g) or blue light (b, d, f, h) using the GFP filter set (EX BP 470/40, BS FT 495, EM LP 550). (PNG 3830 kb) [file 12896_2018_477_MOESM1_ESM.png]

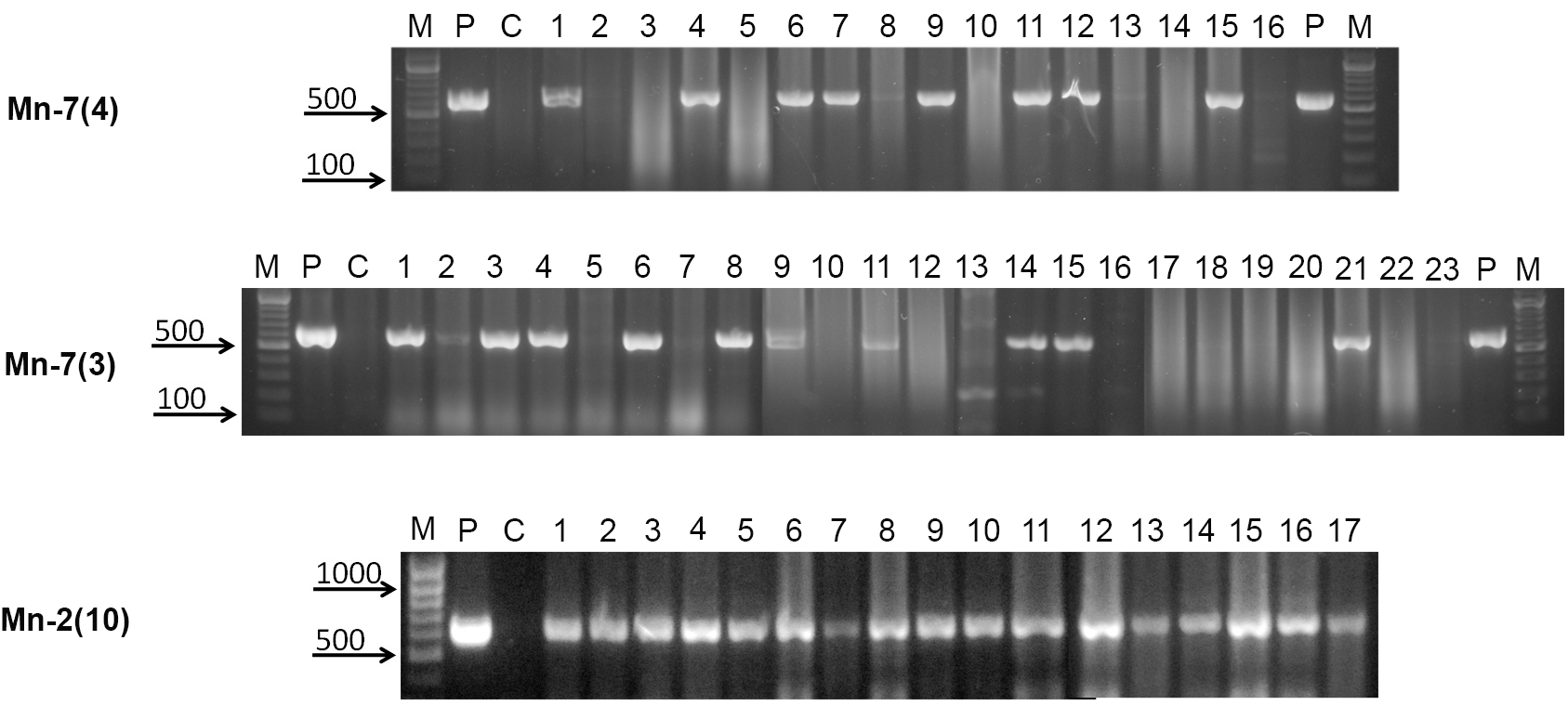

Supplement: Supplementary file 2 — Figure S2. Inheritance of the gfp gene in T2 progeny of transgenic einkorn, assessed by PCR. Examples of a 1:1 ratio segregation (T2 progeny of T1 plants Mn-7(3) and Mn-7(4)) and homozygous transgene inheritance (T2 progeny of T1 plant Mn-2(10)); Lane P, plasmid control, Lane C, untransformed einkorn plant; Lane numbers 1–23, T2 progeny plants. (PNG 421 kb) [file 12896_2018_477_MOESM2_ESM.png]
